# Analysis of Floral Volatile Components and Antioxidant Activity of Different Varieties of *Chrysanthemum morifolium*

**DOI:** 10.3390/molecules22101790

**Published:** 2017-10-23

**Authors:** Lu Yang, Aobulikasimu Nuerbiye, Ping Cheng, Jin-Hui Wang, Hong Li

**Affiliations:** 1 Xinjiang Academy of Forestry, Urumqi 830000, China; yanglukitty127@163.com (L.Y.); chengping1985@163.com (P.C.); 2 Economic forest product quality inspection and testing center of the State Forestry Administration (Urumqi), Urumqi 830052, China; 3 Xinjiang Medical University, Urumqi 830000, China; xjlky_2@163.com (A.N.); tcm_shz@aliyun.com (J.-H.W.)

**Keywords:** *Chrysanthemum morifolium*, ATD-GC/MS, antioxidant activity, principle component analysis (PCA), cluster analysis

## Abstract

This study investigated the volatile flavor compounds and antioxidant properties of the essential oil of chrysanthemums that was extracted from the fresh flowers of 10 taxa of *Chrysanthemum morifolium* from three species; namely *Dendranthema morifolium (Ramat.)* Yellow, *Dendranthema morifolium (Ramat.)* Red, *Dendranthema morifolium (Ramat.)* Pink, *Dendranthema morifolium (Ramat.)* White, *Pericallis hybrid* Blue, *Pericallis hybrid* Pink, *Pericallis hybrid* Purple, *Bellis perennis* Pink, *Bellis perennis* Yellow, and *Bellis perennis* White. The antioxidant capacity of the essential oil was assayed by spectrophotometric analysis. The volatile flavor compounds from the fresh flowers were collected using dynamic headspace collection, analyzed using auto thermal desorber–gas chromatography/mass spectrometry, and identified with quantification using the external standard method. The antioxidant activities of *Chrysanthemum morifolium* were evaluated by DPPH and FRAP assays, and the results showed that the antioxidant activity of each sample was not the same. The different varieties of fresh *Chrysanthemum morifolium* flowers were distinguished and classified by fingerprint similarity evaluation, principle component analysis (PCA), and cluster analysis. The results showed that the floral volatile component profiles were significantly different among the different *Chrysanthemum morifolium* varieties. A total of 36 volatile flavor compounds were identified with eight functional groups: hydrocarbons, terpenoids, aromatic compounds, alcohols, ketones, ethers, aldehydes, and esters. Moreover, the variability among *Chrysanthemum morifolium* in basis to the data, and the first three principal components (PC1, PC2, and PC3) accounted for 96.509% of the total variance (55.802%, 30.599%, and 10.108%, respectively). PCA indicated that there were marked differences among *Chrysanthemum morifolium* varieties. The cluster analysis confirmed the results of the PCA analysis. In conclusion, the results of this study provide a basis for breeding *Chrysanthemum* cultivars with desirable floral scents, and they further support the view that some plants are promising sources of natural antioxidants.

## 1. Introduction

*Chrysanthemum (Chrysanthemum morifolium* Ramat.) belongs to the Asteraceae family of leading ornamental species, second only to the rose in terms of its market value [1]. *Dendranthema morifolium (Ramat.)* and *Pericallis hybrida*, called Qiuju and Fuguiju in China, are widely used in South China, either as a fragrant floral tea or as an anti-inflammatory herb in Traditional Chinese Medicine. They have been reported to possess antibacterial, antifungal, antispirochetal, anti-inflammatory, and antioxidant activities [2]. The flavonoids, alkaloids, and sesquiterpene lactones are thought to contribute to the pharmacological activities of *Chrysanthemum morifolium* [2,3,4]. A recent report indicated that the flavonoids in the extracts of *Chrysanthemum morifolium* protected the brain, liver, and kidney against lead-induced oxidative damage in mice. Moreover, the extracts provided significant protection against cerebral ischemia and reperfusion injury in rats through their antioxidant effect [5,6].

*Chrysanthemum* species have also been proposed as a potential ingredient for herbal cosmetics for their tyrosinase inhibitory activity, which has been associated with antioxidant activation, whitening, and moisturizing effects [7,8,9].

*Bellis perennis* L. (Asteraceae), called Nvwangju, is an herbaceous perennial plant known as a traditional wound herb. It has been used for the treatment of bruises, broken bones, wounds, headaches, the common cold, stomach aches, eye diseases, eczema, skin boils, gastritis, diarrhea, bleeding, rheumatism, inflammation, and infections of the upper respiratory tract in traditional medicine [10].

Automatic thermal desorption (ATD) is a valuable method for the fractionation of plant volatile components that can be carried out online with gas chromatography (GC) analysis. Thermal desorption can present some advantages since it substantially simplifies analyses (there is no need for a concentration step after sampling) and it increases sensitivity (a large part of the pre-concentrated material may be recovered for determination), and detection limits and background noise are lower because of the disappearance of solvent components [11,12,13]. The use of coupled GC-MS affords additional qualitative information, which is of special interest for plant species whose composition has not been previously studied. Some examples of the application of ATD coupled to GC-MS for the identification and characterization of volatile components of plants of different families are given [14]. ATD-GC/MS is a fast, simple, and convenient method to analyze volatile compounds in flowers [15]. ATD-GC/MS has also been successfully applied to detection of pesticide residues [16]. Therefore, we investigated the feasibility of ATD-GC/MS as a tool to predict the aromatic components present in *Chrysanthemum morifolium*.

Recently, plants were studied on numerical taxonomic classification using multivariate data based on their morphological, biochemical, and molecular characteristics [17,18]. In particular, on the basis of the chemical components, like the volatile compounds in plants, multivariate analyses, such as principal component analysis (PCA) and cluster analysis, have been performed [19,20].

PCA used in the present study is essentially that described by Cooley and Lohnes in which a correlation matrix with unit values in its principal diagonal is used as input and the variable-vectors are standardized. Each vector, thus, has zero mean and unit variance and can be taken as being of unit length [21]. The selection of the optimum number of reference axes is necessary to explain the relationships between any particular set of variables. Different workers [22,23] have taken different approaches. Kaiser has suggested that the number of the axes should be restricted to the number of eigenvalues, which are greater than unity and the one used in the present study [24].

Cluster analysis is a type of multivariate analysis that divides data with close similarities into groups (clusters) that are meaningful and useful. If meaningful groups are the goal, then the clusters should capture the natural structure of the data. Therefore, as a technique for grouping clusters of similar traits based on the diverse characteristics of certain entities or subjects, cluster analysis can be utilized in situations where there are no clear or known classification criteria [17]. However, little research has been performed on the *Chrysanthemum* species, including the classification of species based on volatile compounds.

Antioxidants have been intensively studied in pharmaceutical and dermatological fields to prevent or treat disorders related to oxidative stress. In the past, antioxidants have also been used in the food industry to protect against the deterioration of food and in the cosmetic industry to delay or prevent skin aging. Free radicals and reactive oxygen species (ROS) are reported to be associated with several diseases, including inflammation [23] and aging and age-related diseases [25]. Importantly, free radical damage on the skin caused by ROS and UV-irradiation stress plays a key role in photoaging [26,27].

The present study was conducted to obtain clear inter-species classification by using multivariate analysis methods, such as PCA and cluster analysis, as well as by analyzing and comparing the volatile compounds of 10 taxa of three *Chrysanthemum* species. Moreover, the antioxidant activities of the three species were evaluated by DPPH and FRAP assay.

## 2. Results

### 2.1. Optimization of ATD Parameters

**Figure 1:**
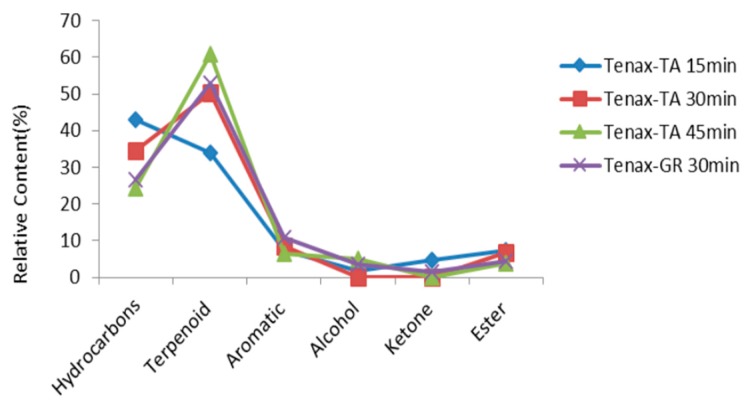
Percentage of alcohols, hydrocarbons, and other compounds released from *Dendranthema morifolium (Ramat.)* Yellow (**No.1**) in dependence of sampling times and absorbents.

The duration of desorption is an important parameter and its optimization can improve desorption efficiency and sensitivity. Therefore, three desorption times are selected for testing: 15, 30, and 45 min. As shown in Figure 1, a duration of desorption of 30 min, 18 compounds were detected and the content of hydrocarbons, aromatics, esters were relatively high. While for 45 min, 15 compounds were detected and the content of all compounds were low except the content of terpenoids. This phenomenon can be explained by the low volatility in addition to their thermal sensibility [28]. Therefore, a 30-min duration was confirmed as the optimization time condition. Tenax-TA and Tenax-GR are polymeric materials (styrene polyvinylbenzene) that are commonly used as an adsorbent for volatile compounds, especially for use with high temperatures. As can be seen in Figure 1, there is no distinctive difference, so the entire experiment path used Tenax-TA as an adsorbent for volatile compounds and the sampling time of 30 min.

### 2.2. Comparison of Antioxidant Activities

Two common in vitro assays were used to evaluate the antioxidant activities, and the results showed that the three species had significantly different antioxidant activity depending on the assay used (Table 1). In the DPPH assay, all of the extracts showed good radical scavenging activity. Samples of *Dendranthema morifolium* had a lower mean EC50 than that of *Bellis perennis* and a higher mean EC50 than that of *Pericallis hybrida*, but no significant difference was found (*p* > 0.05). In the FRAP assay, the antioxidant activity of *Bellis perennis* was significantly stronger than that of *Dendranthema morifolium* and *Pericallis hybrida* (*p* < 0.05) (Table 1). The results indicated that essential oil extracted from *Chrysanthemum morifolium* exhibited DPPH free radical scavenging activity, and therefore it could be applied as an antioxidant agent.

### 2.3. Analysis of Volatile Components and Release Quantity in Different Varieties of Chrysanthemum

The presence, yield, and composition of secondary metabolites, such as volatile oils in plants, can be affected by several factors, including physiological variations, environmental conditions, geographic variations, genetic factors, and evolution [29].

**Figure 2:**
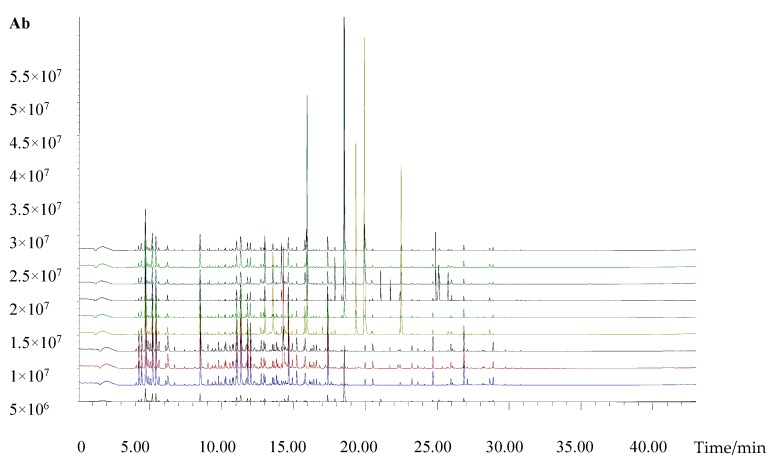
Typical total ion chromatogram of the volatile compounds in *Chrysanthemum morifolium* flowers. From top to bottom, *Dendranthema morifolium* (Ramat.) Yellow (**No.1**), *Pericallis hybrida* Blue (**No.5**), *Pericallis hybrida* Pink (**No.6**), *Pericallis hybrida* Purple (**No.7**), *Dendranthema morifolium* (Ramat.) Red (**No.2**), *Dendranthema morifolium* (Ramat.) Pink (**No.3**), *Dendranthema morifolium* (Ramat.) White (**No.4**), *Bellis perennis* White (**No.10**), *Bellis perennis* Pink (**No.8**), and *Bellis perennis* Yellow (**No.9**), respectively.

A typical GC-MS total ion chromatogram (TIC) of the volatile chemical profile of *Chrysanthemum* is shown in Figure 2.

The volatiles of the 10 taxa are listed in Table 2. GC-MS analysis detected 36 compounds in the floral scent of *Chrysanthemum*, which were identified by their mass spectra and RI. Based on the classification of aromatic compounds of *Chrysanthemum*, volatile compounds were categorized as: terpenoid compounds, hydrocarbons, aromatic compounds, alcohols compounds, and flavor compounds. Hydrocarbons and terpenoids predominated, while the other compounds were typically present in smaller amounts. The 36 detected compounds included 10 hydrocarbons, 10 terpenoids, 4 aromatics, 4 alcohols, 3 ketones, 3 esters, 1 aldehydes, and 1 ether (Table 2). Camphor, *n*-hexane, ethyl acetate, 1,3,5-cycloheptatriene, ethyl benzene, o-xylene, eucalyptol, nonane, alpha-Pinene, and pentadecane were the most common components, and camphor, n-hexane, o-xylene, and eucalyptol were present at particularly high levels.

**Figure 3:**
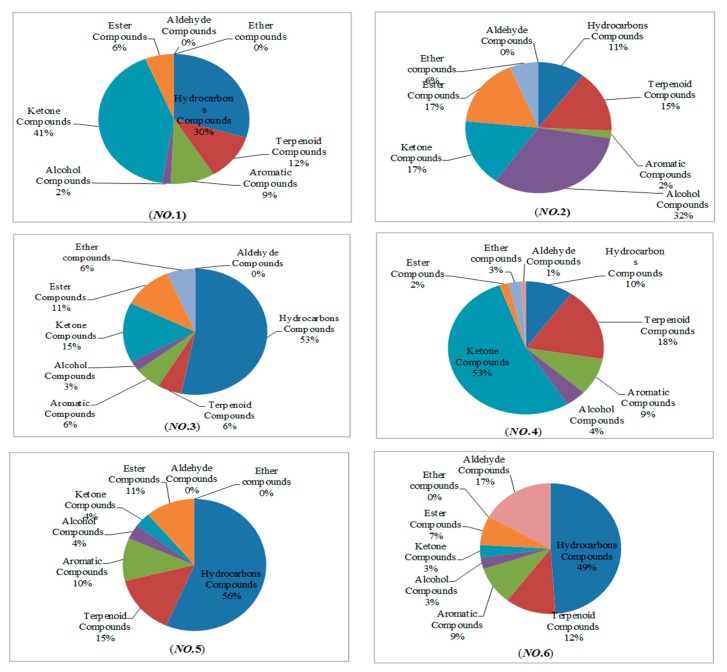

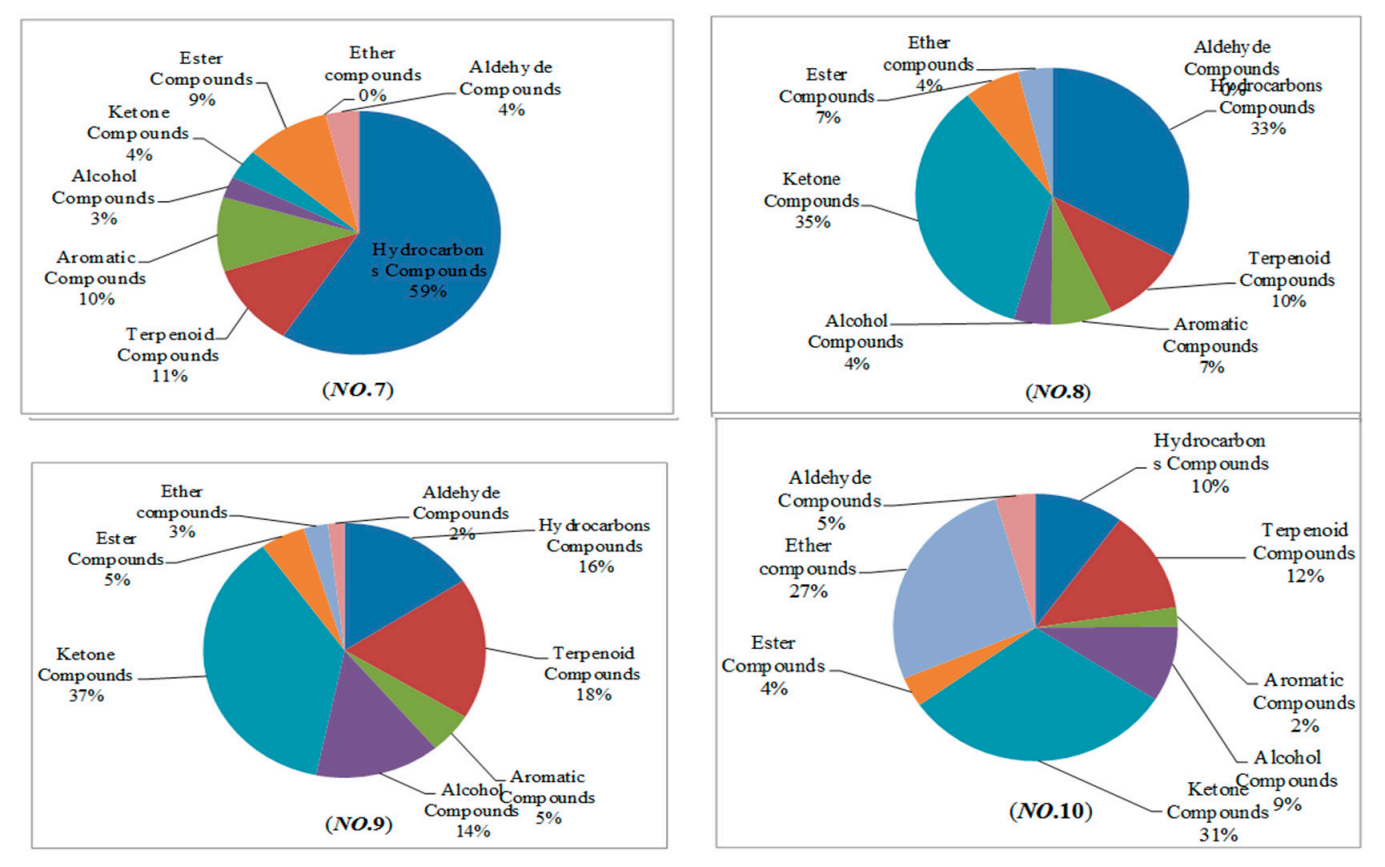
Percentage of terpenoids, hydrocarbons, and other compounds released from fully open *Chrysanthemum* samples of flowers.

The volatile compound contents varied markedly among the 10 taxas. In **Nos. 1, 4, 6, 8, 9,** and **10**, camphor (4.19 μg, 1.29 μg, 9.53 μg, 2.70 μg, 3.76 μg, and 4.06 μg) was the highest component. In **No.2,** endo-Borneol (6.01 μg) was the most abundant, and it was only detected in this cultivar. In **No.6**, the content of the volatile compound benzaldehyde (2.67 μg) was the highest component. Even though camphor was found to be the main volatile compound in the three species, the other volatile compounds were reported differently according to the taxa. There were seven same compounds in the ten taxa. As shown in Figure 3, the floral volatile components were significantly different among *Chrysanthemum* samples. The amount of terpenoids (51%) was the highest in **No.1. No.2** contained 32% alcohols, 17% ketones, and 15% terpenoids. **No.3** contained 53% hydrocarbons and 15% terpenoids. **No.4** contained 70% terpenoids, 10% hydrocarbons, and 9% aromatics. **No.5** contained 56% hydrocarbons, 15% terpenoids, and 11% esters. **No.6** contained 49% hydrocarbons, 18% aldehydes, and 12% terpenoids. ***No*.7** contained 59% hydrocarbons, 11% terpenoids, and 10% aromatics. **No.8** contained 45% terpenoids, 33% hydrocarbons, and 7% aromatics. **No.9** contained 54% terpenoids, 16% hydrocarbons, and 14% alcohols. ***No*.10** contained 43% terpenoids, 10% hydrocarbons, and 27% ether compounds. From superficial observation of the structures, **Nos. 5**, **6**, and **7** were similar, and **Nos. 8**, **9**, and **10** were similar. This also showed that the content from a species of *Chrysanthemum morifolium* was similar.

### 2.4. PCA of Volatile Compounds

The main component analysis was used to explore the data matrix order; this can help determine the weight (importance) of parameters in the total variability through vector size and loads and respective percent contributions. The PCA results give three significant principal components (eigenvalues >1, represents the main variables), which explain 96.509% of the variation in the data (55.802%, 30.599%, and 10.108%, respectively).

**Figure 4:**
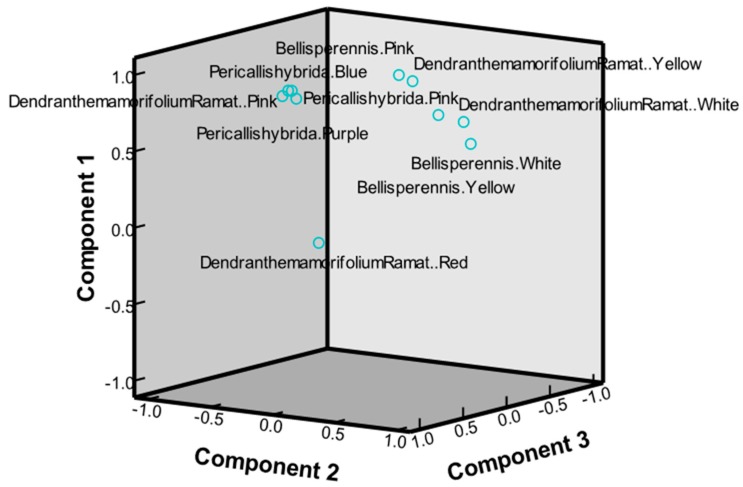
Eigenvalues and their contribution through the PCA using hydrocarbons, terpenoids, aromatic compounds, alcohols, ketone, ether, aldehyde, and ester volatile compounds of 10 taxa of *Chrysanthemum*.

The volatile compounds were evaluated and coded by each graded value to classify the 10 taxa of *Chrysanthemum* from the 3 species. Figure 4 shows the eigenvalues and their contribution through PCA using hydrocarbons, terpenoids, aromatic compounds, alcohols, ketone, ether, aldehyde, and ester volatile compounds of 10 taxa of *Chrysanthemum*. PCA is a method that can be used to identify patterns in a data set and to reduce dimensionality of multivariate data by removing inter-correlations among variables [30,31]. We obtained the eigenvalues of the matrix consisting of a component of the variance and covariance parameter. In the case of the first principal component (PC1), the eigenvalue of each of the characteristics was 5.580, thereby showing a 55.802% contribution to the total variation. The second and third PCs had eigenvalues of 3.060 and 1.011, respectively, and the degree of contribution to the total variation was 30.599% and 10.108%, respectively.

In addition, to estimate the characteristics of the various volatile compounds, correlations between the individual PCs were analyzed. PC1 (55.802% of variance) was accounted for by variables with the highest loads: hydrocarbons (1.676), terpenoids (1.535) compounds, ketones (−0.713), and aldehyde compounds (−0.674). PC2, which explained 30.599% of the variation, was accounted for by the following variables: terpenoids (1.535), alcohols (0.403), and hydrocarbons (−1.736). PC3, which explained 10.108% of the variation, was accounted for by the following variables: alcohols (1.863) and aldehyde compounds (−1.274). This can be interpreted as a gradient of variability among variables; positive signs mean that variables increase together, while a negative sign means that as one variable increases, the other variable decreases. It should also be noted that the sign of the terpenoids variable is the opposite of those of analysis doses because these are derived from the former (they are reciprocals).

As a result of arranging the values of the first three PCs on a 3-dimensional scatter diagram for 10 taxa of the *Chrysanthemum* species, these were categorized into three groups (Figure 4): *Pericallis hybrida* Blue (**No.5**), *Pericallis hybrida* Pink (**No.6**), *Pericallis hybrida* Purple (**No.7**), and *Dendranthema morifolium (Ramat.)* Pink (**No.3**) for the first group; *Dendranthema morifolium (Ramat.)* White (**No.4**), *Bellis perennis* White (**No.10**), *Bellis perennis* Pink (**No.8**), *Bellis perennis* Yellow (**No.9**), and *Dendranthema* *morifolium (Ramat.)* Yellow (**No.1**) for the second group; and *Dendranthema morifolium (Ramat.)* Red (**No.2**) for the third group, respectively. In combination with the PCA, different species of *chrysanthemum* have differences in chemical composition. 

### 2.5. Cluster Analysis of Compositions of Volatile Compounds

**Figure 5:**
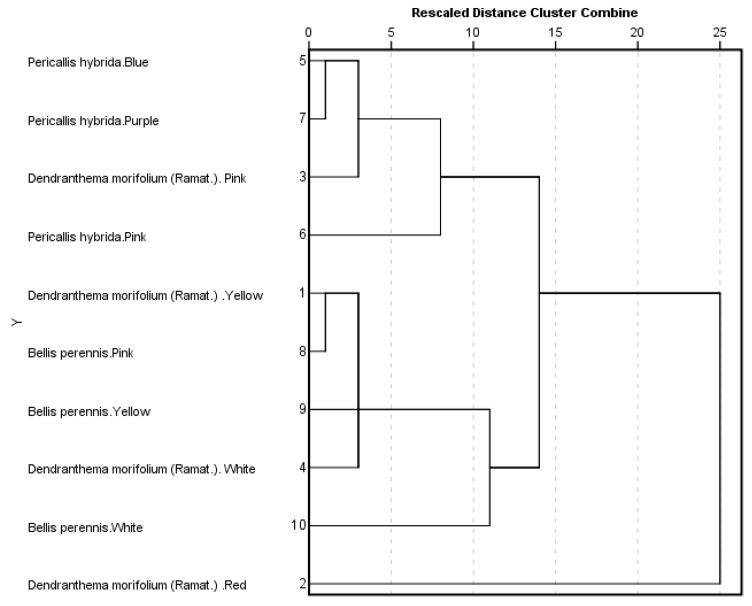
Dendrogram from volatile components of 10 taxa of *Chrysanthemum morifolium* based on Ward’s minimum variance clustering method.

To compare volatile compound compositions among the 10 taxa of *Chrysanthemum* from 3 species, we performed a hierarchical cluster analysis based on the contents of the 36 aromatic volatile compounds. We used Ward’s method for between-group linkage and the squared Euclidean distance between clusters as a proximity measurement. The 10 *Chrysanthemum* formed three clusters in the dendrogram (Figure 5): *Pericallis hybrida* Blue (**No.5**), *Pericallis hybrida* Pink (**No.6**), *Pericallis hybrida* Purple (**No.7**), and *Dendranthema morifolium (Ramat.)* Pink (**No.3**) for cluster I; *Dendranthema morifolium (Ramat.)* White (**No.4**), *Bellis perennis* White (**No.10**), *Bellis perennis* Pink (**No.8**), *Bellis perennis* Yellow (**No.9**), and *Dendranthema morifolium* (Ramat.) Yellow (**No.1**) for cluster II; and *Dendranthema morifolium (Ramat.)* Red (**No.2**) for Cluster III, respectively. Cluster analysis can obtain a variety of classification results according to the scale. The PCA according to the result of principal component scores got only one classification. The PCA and cluster analysis are consistent, and can classify all 10 *Chrysanthemum* taxa from three species.

## 3. Discussion

We investigated for the first time the volatile flavor compounds and antioxidant properties of essential oil that was extracted from fresh flowers, namely *Dendranthema morifolium* (Ramat.) Yellow, *Dendranthema morifolium* (Ramat.) Red, *Dendranthema morifolium* (Ramat.) Pink, *Dendranthema morifolium* (Ramat.) White, *Pericallis hybrida* Blue, *Pericallis hybrida* Pink, *Pericallis hybrida* Purple, *Bellis perennis* Pink, *Bellis perennis* Yellow, and *Bellis perennis* White. *Dendranthema morifolium* (Ramat.), *Pericallis hybrid*, and *Bellis perennis*. All had antioxidant activity, but our study had limitations so we were not able to further define the details of the bioactivity that is responsible for the observed effects. There were 36 volatile compounds that were identified with 8 functional groups: hydrocarbons, terpenoids, aromatics, alcohols, ketones, ethers, aldehydes, and esters. In **Nos. 1**, **4**, **6**, **8**, **9**, and **10**, the content of camphor (4.19 μg, 1.29 μg, 9.53 μg, 2.70 μg, 3.76 μg, and 4.06 μg) was the highest component. In **No.2**, the content of endo-Borneol (6.01 μg) was most abundant, and it was only detected in this cultivar. In **No.6**, the content of the volatile compound benzaldehyde (2.67 μg) was the highest component. Thus, we determined that the chemotaxonomy technique using the compositions of the volatile compounds of the *Chrysanthemum* species would be useful in the classification and identification of the verified inter-species differences. Finally, this study provides the basic materials needed for species selection and cultivation of *Chrysanthemum* species, which are useful for food, cosmetics, and medicine, further supporting the view that the flowers are promising sources of natural antioxidants. The underlying antioxidant mechanisms and potential side effects of flowers also warrant further exploration and experimentation.

## 4. Materials and Methods

### 4.1. Plant Materials

The 10 varieties of *Chrysanthemum morifolium* used in this study were purchased from local markets and maintained by laboratory personnel in the nursery at Xinjiang Academy of Forestry, (Urumqi, China) (Table 3). They were identified by Associate Professor Zhiyou Guo (Department of Life Sciences, Qiannan Normal University for Nationalities, Duyun, China). Alpha-Pinene standard solution (>99.9% purity) was diluted by methanol (HPLC purity) to the concentration range of 1 μg/mL–10 μg/mL. Then, a microliter injector (Anting micro injector factory, Shanghai, China) was used to get 0.5 μL standard solution injected into Tenax TA (PerkinElmer, Boston, MA, USA); it was plugged with glass cotton in order to stop the alpha-Pinene from evaporating. Each concentration was prepared 3 times for the test.

### 4.2. Extraction of Volatile Flavor Compounds

Simultaneous steam distillation (SDE) was performed following Schultz’s extraction method to extract the volatile flavor compounds [32]. Two grams of the freeze-dried leaf sample and 500 mL of distilled water were added to one of the flasks, and heated-reflux was performed for 2 h on a 100 °C heating mantle. Meanwhile, the essential oils were extracted by adding 50 mL of pentanediethyl ether mixture in the other flask and heated-refluxing to 40 °C. The extracted volatile flavor compounds were dehydrated using anhydrous sodium sulfate. Afterwards, they were filtered using filter paper and concentrated using 99.9% nitrogen gas. Finally, the concentrated compounds were dissolved in 100 μL of acetone, and they were analyzed using gas chromatography/mass selective detector (GC/MSD) as below.

### 4.3. Sampling Collection

A single fresh flower from *Chrysanthemum morifolium* was collected in a sampling bag (355 mm × 355 mm, Reynolds, Jefferson, MO, USA). The sampling collection steps included: (a) an air sampler was used to remove the air in the sampling bag; (b) an atmosphere sampler was used to fill the air passed through the activated carbon into the sampling bag; (c) When the content of the air in the sampling bag was close to three-quarters filled, the emission volatile matter from *Chrysanthemum morifolium* was collected by active sampling onto the sorbent Tenax TA. Tenax-TA was chosen as it corresponds to a polymeric material (styrene polyvinylbenzene); it is commonly used as an adsorbent for semi-volatile compounds, especially for use with high temperature. The entire gas path used tasteless silicone tubing. The sampling time was fixed at 10:00–12:00 a.m. The sampling instrument volume flow rate was 100 mL min^−1^, with different sampling times (15, 30, and 45 min). Each variety was sampled 3 times during the same testing session. Empty bags served as the control samples.

### 4.4. Analytical Instrumentation

For the ATD, a two-stage desorption was adopted to reduce the component bandwidths and improve the efficiency of the chromatographic separation. Thermal primary desorption of the sampling tubes was carried out at 250 °C with a helium flow rate of 25 mL min^−1^ for 20 min in order to maintain conditions strictly similar to the online sampling. The outlet split was also fixed to 5 mL min^−1^. The cold trap was maintained at −30 °C. Again, during secondary desorption, the cold trap was rapidly heated from −30°C to 300 °C, and it was maintained at this temperature for 5 min. Analytes were then injected onto the capillary column via a transfer line that was heated to 250 °C. The outlet-split flow was adjusted for high-resolution capillary column to 10 mL/min, leading to a fraction of 50% of the sample entering the column [33].

ATD–GC–MS: automatic thermal desorption (ATD 350, PerkinElmer Corp., Norwalk, CT, USA) with gas chromatography (GC) coupled with a mass selective detector (MSD). The chromatograph was a 7890A Network GC System interfaced with a 5975C Network MSD (Agilent Technologies, Santa Clara, CA, USA), and the capillary column was a Thermo TR-5ms (60 m × 0.25 mm; film thickness of 0.25 μm). The MS detector provided acquisition in the full-scan mode or selected ion monitoring (SIM) mode. The ion energy of electron impact ionization was 70 eV, with the ion source temperature set to 250 °C. Helium was used as the carrier gas at 1.5 mL/min (with regulated constant flow). The GC oven was set at 40 °C for 2 min, followed by 6 °C/min to 280 °C, with a final extension for 3 min at 280 °C, and a total acquisition program of 45 min. Mass spectral data were acquired over a mass range of 29–500 amu for the full-scan mode. The qualitative identification of targeted compounds was based on retention times. Quantification of the extracted ions was performed using the external standard method.

### 4.5. Antioxidant Capacity Analysis

DPPH assay was performed according to the Brand–Williams method [34], which was slightly modified by Kim et al. [35]. In brief, 2 mL of *Chrysanthemum morifolium* extract solution and 2 mL of DPPH (200 μmol/L) were mixed and the absorbance was measured after 30 min at 517 nm at room temperature using an ultraviolet spectrophotometer and the effective concentration. The effective concentration (EC50) value was determined for the antioxidants. The FRAP assay was determined according to Li’s method [36]. The FRAP assay measures the ability of the antioxidants in *Chrysanthemum morifolium* extracts to reduce the ferric tripyridyl-triazine (Fe^3+^-TPTZ) complex to the blue-colored ferrous form (Fe^2+^), which absorbs light at 593 nm. Briefly, a sample extract (0.5 mL) was mixed with 3.0 mL of ferric-TPTZ reagent and added to 10 mL PV tubes. The tubes were incubated at 37 °C for the duration of the reaction. The absorbance was read at 593 nm at 75 min with an ultraviolet spectrophotometer (UV-2600/2700, Shimadzu, Japan).

### 4.6. Statistical Analysis

Based on the results of the GC/MSD analysis of the volatile compounds in the 10 taxa of *Chrysanthemum* species, ANOVA and multivariate analyses were performed using IBM’s SPSS 19.0 software (IBM SPSS Institute, ver. 19.0, Chicago, IL, USA).

## Figures and Tables

**Table 1:** Antioxidant activity values for *Chrysanthemum morifolium*.

**Samples**	**1**	**2**	**3**	**4**	**5**
DPPH IC50 (mgml^−1^)	0.089 ± 0.003	0.109 ± 0.001	0.091 ± 0.008 **	0.097 ± 0.007	0.113 ± 0.011
FRAP C (Fe^2+^) (mmolg^-1^) ³	2.300 ± 0.011 *	2.810 ± 0.012 *	2.110 ± 0.021 *	2.560 ± 0.019 *	3.170 ± 0.017 *
**Samples**	**6**	**7**	**8**	**9**	**10**
DPPH IC50 (mgml^−1^)	0.108 ± 0.009	0.119 ± 0.0010	0.072 ± 0.003	0.080 ± 0.005	0.082 ± 0.001
FRAP C (Fe^2+^) (mmolg^−1^) ³	3.090 ± 0.022 **	3.209 ± 0.019 *	1.870 ± 0.009 *	1.990 ± 0.010 *	1.807 ± 0.007 *

Note: Values for *Chrysanthemum morifolium*, which differ and were statistically significantly, are marked with “*”. Analysis of variance was performed between various groups of *Chrysanthemum morifolium*. * *p* < 0.05. ** *p* < 0.01. Antioxidant activity expressed as mmol FeSO_4_ equivalents per 1 g sample.

**Table 2:** Volatile compounds released from the flowers of 10 varieties of *Chrysanthemum morifolium*.

NO.	Retention Time (min)	Compounds	Molecular Formula	Relative Molecular Mass	Content (μg)										MS Fragment Peaks
					1	2	3	4	5	6	7	8	9	10	
**1**	4.217	Ethanol	C_2_HH_5_O	46.0	nd	nd	0.369	nd	0.567	0.463	0.467	nd	nd	nd	31,46,60
**2**	4.412	Acetone	C_3_H_6_O	58.0	0.222	nd	0.472	nd	0.574	0.502	0.653	nd	0.141	0.133	31,38,41,58
**3**	4.699	Methylene chloride	CH_2_C_l2_	84.0	1.13	0.731	2.88	0.861	2.772	2.348	2.854	0.904	0.751	0.677	35,49,84,88
**4**	4.856	Pentane, 2-methyl-	C_6_H_14_	86.1	nd	nd	0.459	nd	0.190	0.195	0.266	nd	nd	nd	41,56,71,86
**5**	5.175	n-Hexane	C_6_H_14_	86.1	0.705	0.421	2.19	0.433	1.53	1.35	1.72	0.557	0.357	0.309	29,41,43,57,60,86
**6**	5.424	Ethyl Acetate	C_4_H_8_O_2_	88.1	0.664	0.291	1.54	0.347	1.56	1.15	1.53	0.509	0.310	0.252	29,43,61,70,88
**7**	5.63	Cyclohexane	C_6_H_12_	84.1	nd	nd	0.263	nd	0.234	0.220	0.284	nd	nd	nd	41,56,60,69,84
**8**	6.236	Benzene	C_6_H_6_	78.0	0.173	nd	0.342	nd	0.252	0.263	0.277	nd	nd	nd	31,39,41,43,50,52,56,78
**9**	8.498	1,3,5-Cycloheptatriene	C_7_H_8_	92.1	0.615	0.397	0.754	0.443	1.58	1.41	1.45	0.586	0.299	0.239	39,51,65,91
**10**	11.037	Ethylbenzene	C_8_H_10_	107	0.275	0.163	0.254	0.197	0.644	0.591	0.592	0.283	0.136	nd	39, 51, 65, 77, 91, 106
**11**	11.795	Nonane	C_9_H_20_	128	0.231	0.172	0.262	nd	0.703	0.634	0.731	0.234	0.118	nd	41,57,71,85,99,128
**12**	11.995	o-Xylene	C_8_H_10_	106	0.282	0.191	0.227	0.173	0.765	0.670	0.741	0.275	0.132	nd	39,51,65,75,91,103,109
**13**	12.753	Linalye Acetate	C_12_H_20_O_2_	196	nd	nd	nd	nd	0.202	nd	nd	nd	nd	nd	39,43,57,71,113
**14**	13.002	alpha.-Pinene	C_10_H_16_	136	0.213	0.577	0.109	0.253	0.198	0.159	0.153	0.199	0.631	0.715	41,79,93,98,121,136
**15**	13.57	Camphene	C_10_H_16_	136	nd	1.439	nd	nd	nd	nd	nd	nd	0.437	0.207	29,43,57,70,98
**16**	13.828	1-Decene	C_10_H_20_	140	nd	nd	nd	nd	0.204	0.190	nd	nd	nd	nd	29,41,57,71,83,112,125
**17**	14.29	Benzaldehyde	C_7_H_6_O	106	nd	nd	nd	0.180	nd	2.67	0.629	nd	0.199	0.612	51,77,106
**18**	14.382	beta.-Pinene	C_10_H_16_	136	0.193	nd	nd	nd	nd	nd	nd	nd	nd	nd	41,69,79,93,121,136
**19**	14.636	Decane	C_10_H_22_	142	0.407	0.293	0.511	0.243	1.20	1.02	1.20	0.415	0.215	0.186	43,57,71,85,99,113,142
**20**	15.232	Decane, 4-methyl-	C_11_H_24_	156	nd	nd	nd	nd	0.305	0.288	0.307	nd	nd	nd	53,57,71,84,113
**21**	15.789	D-Limonene	C_10_H_16_	136	nd	0.432	nd	nd	0.326	0.269	0.336	nd	0.377	0.159	41, 53, 68, 79, 93, 107, 121, 136
**22**	15.946	Eucalyptol	C_10_H_18_O	154	nd	1.177	0.868	0.477	nd	nd	nd	0.312	0.295	3.60	65,79,93,121,136
**23**	17.37	Undecane	C_11_H_24_	156	0.449	0.307	0.539	0.262	1.26	1.11	1.33	0.428	0.228	0.193	43,57,71,85,98,112,127,156
**24**	17.879	cis-Ocimene	C_10_H_16_	136	0.214	nd	nd	0.832	nd	nd	nd	nd	0.148	0.177	27,41,53,67,79,93,105,121,136
**25**	18.528	Camphor	C_10_H_14_O	150	4.19	nd	1.29	9.53	nd	nd	nd	2.70	3.76	4.06	39,65,79,91,107,122,150
**26**	19.34	Bicyclo[3.1.1] hept-2-en-6-one, 2,7,7-trimethyl-	C_10_H_16_O	152	nd	3.08	0.350	nd	nd	nd	nd	nd	nd	nd	41,55,69,81,95,108,152
**27**	19.93	endo-Borneol	C_10_H_18_O	154	nd	6.01	nd	nd	nd	nd	nd	0.347	1.52	1.23	41,55,67,95,110,139
**28**	21.072	verbenol	C_10_H_16_O	152	0.204	nd	nd	0.481	nd	nd	nd	nd	nd	nd	43,81,91,109,119,134
**29**	21.742	Carveol	C_10_H_16_O	152	nd	nd	nd	0.309	Nd	nd	Nd	nd	Nd	n d	43,79,91,107,119,134
**30**	22.398	Carvone	C_10_H_14_O	150	nd	nd	nd	0.229	Nd	nd	Nd	nd	nd	nd	39,64,82,135,150
**31**	22.485	Bornyl acetate	C_12_H_20_O_2_	196	nd	2.88	nd	nd	nd	nd	nd	nd	0.233	0.235	43,80,95,108,121,136
**32**	24.682	Tetradecane	C_14_H_3_0	198	nd	nd	nd	nd	0.247	0.227	0.308	nd	nd	nd	41,57,71,99,112,127,141,198
**33**	24.883	r-Elemene	C_15_H_24_	204	nd	nd	nd	1.31	nd	nd	nd	nd	nd	Nd	41,53,68,81,93107,121,161,189
**34**	25.099	Thymol	C_10_H_14_O	150	0.283	nd	nd	1.27	nd	nd	nd	nd	0.275	0.314	39,65,79,91,107,135,150
**35**	25.749	Caryophyllene	C_15_H_24_	204	nd	nd	nd	0.421	nd	nd	nd	nd	nd	0.182	41,67,79,91,107,135,150
**36**	26.842	Pentadecane	C_15_H_24_	212	0.247		0.184		0.474	0.372	0.577				41,56,85,99,127,169,212

Note: *Dendranthema morifolium* (Ramat.) Yellow (**No.1**), *Dendranthema morifolium* (Ramat.) Red (**No.2**), *Dendranthema morifolium* (Ramat.) Pink (**No.3**), *Dendranthema morifolium* (Ramat.) White (**No.4**), *Pericallis hybrida* Blue (**No.5**), *Pericallis hybrida* Pink (**No.6**), *Pericallis hybrida* Purple (**No.7**), *Bellis perennis* Pink (**No.8**), *Bellis perennis* Yellow (**No.9**), and *Bellis perennis* White (**No.10**). Nd indicates the compounds that were of too low concentration to be detected.

**Table 3:** *Dendranthema morifolium* (Ramat.), *Pericallis hybrida*, and *Bellis perennis* with different colored flowers.

NO.	Cultivar	Flower Color	Flower Diameter (cm)
**1**	*Dendranthema morifolium (*Ramat.*)* Yellow	Yellow	4.8
**2**	*Dendranthema morifolium (*Ramat.*)* Red	Red	4.5
**3**	*Dendranthema morifolium (*Ramat.*)* Pink	Pink	5.3
**4**	*Dendranthema morifolium (*Ramat.*)* White	White	4.5
**5**	*Pericallis hybrida* Blue	Royal blue	4.2
**6**	*Pericallis hybrida* Pink	Pink	4.2
**7**	*Pericallis hybrida* Purple	Amaranth	4.2
**8**	*Bellis perennis* Pink	Pink flowers with yellow hearts	3.3
**9**	*Bellis perennis* Yellow	Yellow	3.0
**10**	*Bellis perennis* White	White	3.2
